# Evaluation of Visual Disturbances After Mild Traumatic Brain Injury—A One-Year Follow-up Study

**DOI:** 10.1097/HTR.0000000000001010

**Published:** 2024-11-07

**Authors:** Möller Mona-Lisa, Mäki Kaisa, Nybo Taina, Huovinen Antti, Marinkovic Ivan, Melkas Susanna, Johansson Jan

**Affiliations:** **Author Affiliations:** Department of Neurology, University of Helsinki and Helsinki University Hospital, Helsinki, Finland (Mrs Möller, Mr Huovinen, Mr Marinkovic, and Mrs Melkas); Department of Neuropsychology, University of Helsinki and Helsinki University Hospital, Helsinki, Finland (Mrs Mäki and Mrs Nybo); and Department of Clinical Neuroscience, Eye and Vision, Karolinska Institutet, Stockholm, Sweden (Johansson).

**Keywords:** concussion, mild traumatic brain injury, vision assessment, vision evaluation, visual disturbances

## Abstract

**Objective:**

To examine the persistence of visual symptoms in mild traumatic brain injury (MTBI) during the first months after injury. It is important to recognize visual disturbances because they can delay return to normal activities, while they might be simultaneously treated by visual therapy. Here we describe the results from a 1-year follow-up study of visual disturbances after MTBI.

**Participants and Measures:**

The study group comprised 26 patients from the Brain Injury Clinic of the Helsinki University Hospital. Inclusion criterion was a high score (≥21p) on the Convergence Insufficiency Symptom Survey (CISS) at an appointment with a neurologist within 6 months after injury. The patients underwent baseline vision evaluation at 4 months on average and follow-up at 14 months after injury. The evaluation included tests for visual acuity, near point of convergence, convergence facility, near point of accommodation, accommodative facility, motility, heterophoria, binocular vision, dynamic visual acuity, and fusional vergence width at near and far distances. Further assessments included the Rivermead Post Concussion Questionnaire for posttraumatic symptoms, a visual analog scale for visual fatigue, and the Developmental Eye Movement Test for saccadic eye movements.

**Results:**

Both CISS and Rivermead Post Concussion Questionnaire scores improved significantly from baseline to follow-up. The overall level of visual fatigue according to visual analog scale score was lower at follow-up, but the increase in visual fatigue (comparing fatigue before and after assessment session) was significant both at baseline and follow-up. In visual function assessments, spontaneous recovery from baseline to follow-up could be seen in vergence facility and pursuit eye movement but not in near point of convergence, near fusion, distance fusion, heterophoria, and dynamic visual acuity.

**Conclusion:**

The results point out the importance of evaluation of visual disturbances after MTBI. Early detection of these disturbances may provide an opportunity to provide visual therapy.

## BACKGROUND

Research shows that the annual incidence of mild traumatic brain injury (MTBI) is about 1% of 100 000 persons and the median time to recover is about 100 days.^1^ Common symptoms in the acute stage for adults with MTBI are cognitive deficits and visual symptoms. Recovery is reported for most within 3 to 12 months.^2^ A network of over 30 different cortical areas^3^ with more than 300 intracortical visual pathways involves about 50% of the cortex in visual processing.^4^ Visual functions are therefore susceptible to injury after acquired brain injury.^5^ Between 30% and 85% of the patients are estimated to have visual disturbances^6^ after traumatic brain injury.

The frequency of reported visual disturbances varies in different studies after MTBI. A study of adolescents with concussion^7^ found that 69% received 1 or more vision diagnoses after concussion. Eye movement problems in adults are reported to be as high as 90% in concussions or blast injuries.^8^ Persistent visual disturbances remain in about 10% to 20% of MTBI patients.^8^

Visual disturbances commonly include anomalies of accommodation, version eye movements (saccades and pursuit eye movements) and vergence, eye motility disorders, eye teaming, photosensitivity, and ocular health issues.^6^,^8^,^9^ Vision-related tasks such as reading require good accommodative, version, and vergence functions.^5^ It is important to recognize visual disturbances that can affect the return to daily activities.^10^ The visual disturbances may be targeted with visual therapy, which has also been promising after MTBI.^11^

However, previous documentation on visual disturbances after traumatic brain injury comes mainly from studies on brain injuries more severe than MTBI. Little is known about either spontaneous visual recovery after MTBI or which persistent visual disturbances are the most common.

This study’s primary objective was to examine the persistence of visual symptoms 1 year after MTBI. Secondary objectives included evaluation of the Rivermead Post Concussion Questionnaire (RPQ) and the Convergence Insufficiency Symptom Survey (CISS) scale as measurements of subjective visual disturbances and to investigate the spectrum of long-term visual disturbances.

## MATERIALS AND METHODS

### Definition of mild traumatic brain injury

The World Health Organization’s definition for MTBI was used.^12^ It includes one or more of the following criteria:^1^ confusion or disorientation, loss of consciousness for 30 minutes or less, posttraumatic amnesia for less than 24 hours and/or other transient neurological abnormalities such as focal signs, seizure, and intracranial lesion not requiring surgery;^2^ a Glasgow Coma Scale score of 13 to 15 after 30 minutes postinjury or later upon presentation for healthcare. These manifestations of MTBI must not be due to drugs, alcohol, or medications, or caused by other injuries or treatments for other injuries, other problems, or penetrating craniocerebral injury.

### Patients

The participants (*n* = 26) were recruited from the Brain Injury Clinic at the Helsinki University Hospital between 2017 and 2022. All participants underwent brain magnetic resonance imaging (MRI) within 6 months after MTBI, and all MRI scans were evaluated by a board of certified neuroradiologists. The study included participants with MTBI who suffered from visual symptoms captured with the CISS. The population’s age was 18 to 68 years, ie, of working age. Participants were excluded whose visual disturbances were not attributed to injury, an inability to communicate in Finnish, Swedish, or English, an inability to participate due to persistent drug abuse. A neurologist selected the participants in the study based on their CISS score: a score of 21 or more was considered to indicate increased visual symptoms.^13^,^14^ All vision assessments were performed by the same vision therapist (M.M.). The participants did not receive visual therapy during the follow-up.

All included patients gave their written consent. The study was approved by the Ethics Committee of Helsinki University Hospital (dnro 16.08.2017 §154 and 27.11.2019 §192).

### Evaluation and measures

The participants were examined by a neurologist and a vision therapist at the Traumatic Brain Injury Outpatient Clinic, Helsinki University Hospital (HUS). On average, the patients underwent baseline vision evaluation at median 4 (range 1-9) months and follow-up at median 14 (10-26) months after injury.

The CISS^13^,^14^ scale consists of 15 questions about visual symptoms experienced when performing a near task such as reading, writing, or computer work. The symptoms are measured at a 5-point scale: (0) not experienced the symptom, (1) infrequently, (2) sometimes, (3) fairly often, and (4) always. A total score of 21 points or more is considered symptomatic. The CISS scale was translated into Finnish by permission from the author of the original version.

The RPQ^15^ consists of 16 questions that include somatic, emotional and cognitive complaints commonly reported after MTBI. Three out of 16 questions concern visual functions (blurred vision, light sensitivity, and double vision). A 5-point scale from 0 to 4 rating presence and severity of symptoms over the past 24 hours compared to the same symptoms before injury is reported by the respondents. The scale ratings are: not experienced the symptom (0), no more of a problem,^1^ mild problem,^2^ a moderate problem,^3^ and a severe problem.^4^ In our analysis, the total RPQ score also included scale 1 (no more of a problem).

The visual analog scale (VAS)^16^ is a subjective response scale that was used to estimate visual fatigue before and after the vision assessment session. The respondent indicates the level of visual fatigue by selecting a position along a 10-cm line between 2 end points. The participants were asked to put a mark at the line showing the tiredness in their eyes. The end points in the VAS scale used in this research corresponded to no fatigue at all (score 0) and the worst imaginable fatigue (score 10).

The Developmental Eye Movement Test (DEM)^17^,^18^ is used to measure the quality of saccades and eye movement performance. The DEM test comprises 1 pre-test card and 3 test cards. The first 2 cards (A and B) comprise 20 numbers arranged in 2 columns for each card. The third card consists of 80 numbers distributed over 16 horizontal lines. A ratio is calculated by dividing the adjusted horizontal reading time with the adjusted vertical reading time. The test is performed binocularly at near distance, and the patient reads the numbers aloud.

### Clinical measures of visual functions

The evaluation of visual function included eye motility, visual acuity, near point of convergence, convergence facility, near point of accommodation, accommodative facility, fusional vergence width at near and far, heterophoria, dynamic visual acuity (DVA), and saccades (Table 1). The normative standards and criteria for normal function were derived from the literature.^19^–^21^ The Lea number line test was used to test the basic visual acuity and was interpreted by the normative values according to international recommendations. Near point of accommodation was interpreted by the normative values in centimeters according to age.

**TABLE 1:** Visual function and method of examination with criteria for normal function

Visual function	Method of assessment	Criteria for normal function
Visual acuity (decimals)	Visual chart Lea numbers at 40 cm and 3 m	≥0.8 normal with best correction
Near point of convergence, cm	Push-up method using the convergence fixation target on a Royal Air Force Near Point Rule (RNPR)	≤6 cm^16^
Convergence facility (cycles per minute, CPM)	3 base in (BI) 12 base out (BO) prism	Age < 40 y: ≥11CPM^17^Age ≥ 40 y: ≥7 CPM
Near point of accommodation, cm	Push-up method using the near chart target on an RNPR	Age-related mean score; 30 y ≤ 11.5 cm and 40 y ≤ 17.5 cm. Criteria near point of accommodation less than minimum expected according to the Hoffstetter formula (15-0.25 × age)
Accommodative facility, CPM	Spherical flipper ± 1.0 or ± 1.5 diopters	>10 CPM^18^
Fusional vergence width at near and far viewing (prism diopters = PD)	Prism bar, break point	≤27 PD^16^
Pursuit eye movements	Following an object at near distance horizontally and vertically	Imprecise or jerky pursuit eye movements observed repeatedly (>50%)
Dynamic visual acuity (decimals)	Manually rotating head horizontal while patient keeps up the focus on long distance acuity test	Ability to see the same row at long distance chart clearly as without rotation. Three rows lower acuity = deviation
Heterophoria	Cover test	Smooth and swift recovery
Binocular vision (seconds of arc) Saccades	Test for stereoscopic vision: TNOThe Developmental Eye Movement Test	Normative test values Normative test values

### Statistical analysis

Continuous data are presented as a mean with SD (age) or medians with interquartile range (IQR) and range (nonnormally distributed variables), and categorical data are presented as numbers and percentages. Group comparisons between normal and abnormal findings in measurements were performed using Pearson’s χ^2^ test and Fischer exact test. The Phi Coefficient was reported as supplementary information measure for the strength of an association between 2 categorical variables. Continuous variables CISS, RPQ, and VAS were tested with one-way analysis of variance, and the correlations between CISS and RPQ were analyzed using Pearson’s χ^2^ test. We considered *P* values < .05 as statistically significant. Statistical analysis was performed with SPSS version 27 (www.ibm.com/products/spss-statistics).

## RESULTS

### Sample characteristics

The mean age of the patients (*n* = 26) was 46.1 years (±12.6) (range 24-66), for women (*n* = 18) 47.7 years (±12.5) (range 25-66) and for men (*n* = 8) 42.6 years (±12.9) (range 24-61). The most common cause of injury was ground-level fall (*n* = 15). Other causes were struck by an object (*n* = 4), bicycle accident (*n* = 2), traffic accident (*n* = 3), fall from a height (*n* = 1), and abuse (*n* = 1). Brain MRI showed microhemorrhages in 4 of the patients, and no other lesions were detected.

### Symptoms

The most common symptoms according to CISS at baseline, graded as occurring fairly often or always, were complaints about tired (*n* = 21) and uncomfortable (*n* = 19) eyes and blurred vision (*n* = 16). The participants also complained about frequent re-reading (*n* = 13), losing concentration (*n* = 13) while reading, and the feeling of slow reading (*n* = 11). At follow-up, the main symptoms reduced as follows: feeling tired (*n* = 11), uncomfortable (*n* = 6), having blurred vision (*n* = 7), re-reading (*n* = 4), losing concentration (*n* = 4) while reading and feeling of slow reading (*n* = 7).

**Figure 1.**
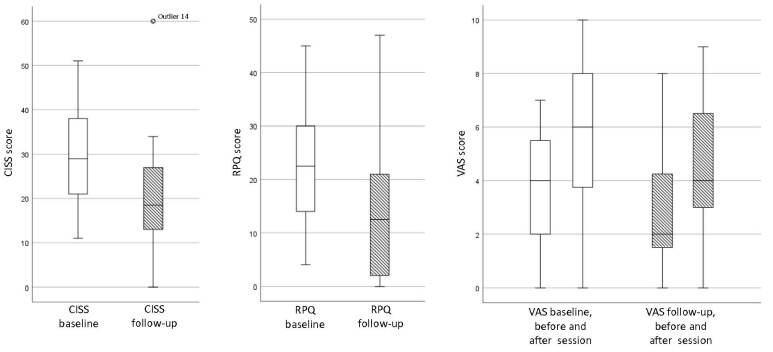
Comparison of symptom scores at baseline and at one-year follow-up.

The median CISS symptom score was at baseline 29 (IQR 20-38, range 11-51), and at follow-up 18.5 (IQR 12-27, range 0-60, *P* < .001) (Figure 1). The median RPQ score was at baseline 22.5 (IQR 13-30, range 4-45, SD 12.6) and at follow-up 12.5 (IQR 2-21, range 0-43, SD 13, *P* < .001) (Figure 1). The CISS and RPQ scores correlated with each other both at baseline (*r*_s_ 0.561, *P* < .003) and at follow-up (*r*_s_ 0.824, *P* < .001).

The median VAS score at baseline indicating self-estimated visual fatigue was 4.0 (IQR 2-6, range 0-7) before assessment session and 6.25 (IQR 3-8, range 0-10) after it. The median VAS score at follow-up was 2.0 (IQR 1-4, range 0-8) before assessment session and 4.0 (IQR 3-7, range 0-9) after it (Figure 1). The increase in VAS score from before to after the assessment session was significant both at baseline (*P* = .002) and at follow-up (*P* < .001), but the overall level of visual fatigue was lower at follow-up.

### Visual function assessment

The main abnormal findings in visual function at baseline were distance fusion (*n* = 24), vergence facility (*n* = 22), heterophoria (*n* = 20), pursuit eye movement (*n* = 19), and near fusion (*n* = 17). The main abnormal findings at follow-up were distance fusion (*n* = 19), heterophoria (*n* = 18), near fusion (*n* = 16), and vergence facility (*n* = 12). In DVA, 7 of 8 had persistent deviant test results at follow-up. All those who had abnormal findings at follow-up had had them already at baseline.

Comparing distribution of normal and abnormal findings at baseline and follow-up, significant improvement was seen in vergence facility (*P* = .004) and pursuit eye movements (*P* < .001) but not in other functions (near point of convergence, near fusion, distance fusion, latent deviation, and DVA; Table 2). The frequency of persistent abnormal findings was high in near fusion (58%), distance fusion (69%), and accommodation (67%).

**TABLE 2:** Comparison of visual functions at baseline and at 1-year follow-up

Visual function	Baseline, *n* = patients		Follow-up, *n* = patients		Statistics	
**Normal**	**Abnormal**	**Normal**	**Abnormal**	**Phi**	***P***
**Vergence facility**	**4**	**22**	**14**	**12**	**0.404**	**.004**
Near point of convergence	18	8	21	5	0.133	.337
Near fusion	9	17	10	16	0.040	.773
Distance fusion	2	24	7	19	0.254	.067
**Pursuit eye movement**	**7**	**19**	**19**	**7**	**0.462**	**<.001**
Dynamic visual acuity	18	8	19	7	0.042	.760
Accommodation (n = 9 < 40y)	4	5	3	6	0.234	.629
Heterophoria	6	20	8	18	0.087	.532

Measure of strength (Phi) and significance (*P*) were analyzed by Pearson’s χ^2^ test and Fischer exact test. Vergence facility = ability to swiftly align the eyes for viewing objects at different distances. Pursuit eye movement = ability to maintain gaze on a moving object.

The median DEM ratio score rose from 1.03 at baseline to 1.13 at follow-up, but the difference was not significant.

Near point of accommodation was measured in 9 pre-presbyopic participants (under age 40 years). Abnormal results at baseline were found in 5 participants. A total of 6 participants (5 previously mentioned plus 1 more) had abnormal results at follow-up. Five of these 6 participants also had remaining abnormal results in vergence facility and distance fusion, and 3 had abnormal DEM ratio. Four of them also had CISS score above 21.

A total of 21 participants had a stereoacuity of 240 seconds of arcs or less (better) at the baseline according to the test for stereoscopic vision (TNO). Two participants failed the test at first assessment, ie, they could not detect the disparity-induced depth clues. They performed normally at follow-up. The improvement in stereoacuity could not be associated with any visual function improvement.

## DISCUSSION

Our 1-year follow-up study after MTBI found that spontaneous recovery was among the reported symptoms according to both the vision specific symptom score, CISS, and the general posttraumatic symptom score, RPQ. The overall level of visual fatigue according to VAS scores was lower at follow-up, but the increase in visual fatigue (comparing fatigue before and after assessment session) was equal at baseline compared to follow-up. Spontaneous recovery in visual function could be seen in the visual function assessments of vergence facility and pursuit eye movement but not in other assessments.

According to our knowledge, self-estimating scales like CISS, RPQ, and VAS have not been previously used in follow-up studies of visual disturbances in MTBI patients. The follow-up studies have instead most often been conducted using only visual function assessments.^5^,^6^ These studies have indicated spontaneous recovery in the majority (80%-90%) of cases. A recent cross-sectional study used RPQ and CISS symptom score to estimate the prevalence of convergence insufficiency and reported significantly higher RPQ score for patients with symptomatic convergence insufficiency (CI) and positive correlation between CISS and RPQ total scores.^22^ The CISS symptom score improved significantly from baseline to follow-up in our study, indicating spontaneous recovery of visual symptoms during the first year after trauma. According to a study by Thiagarajan and colleagues, an improved reading rate also relates to improved CISS score.^23^ It appears that improving oculomotor functions can also improve reading and reduce symptoms in near vision functions.^9^,^24^ CISS is considered as a reliable and valid instrument to clinically measure symptomatic convergence insufficiency in adults with CI. In a study using CISS as a primary outcome measure for 19- to 30-year-old adults with symptomatic CI scored significantly higher than the comparison group with normal binocular vision.^14^

We observed a significant reduction of RPQ score during the first year after MTBI, which is in line with previous documentation. For example, a long-term factor structured study investigating a group of 232 adults 4 years after MTBI found that their RPQ score differed minimally from age- and gender-matched controls.^25^ Only 3 out of 16 questions directly concern visual functions (blurred vision, light sensitivity, and double vision), and therefore, only using RPQ as an initial screening aid detecting visual disturbances in MTBI patients may not be useful. We observed positive correlation between CISS and RPQ scores in line with recent studies.^22^ Using a combination of RPQ and CISS can be an effective combination detecting visual disturbances in MTBI patients.

Concerning visual fatigue, no previous follow-up studies have been identified that focused on this theme. To our knowledge, there is no literature that describes visual fatigue after a vision examination. There are, however, findings of increased visual fatigue after visually demanding tasks (the examination could be considered a visually demanding task) where the fatigue also correlates with measures of binocular function.^24^ Furthermore, there are indications that compensation for fatiguing oculomotor tasks happens through a central nervous system mechanism rather than through peripheral muscular mechanisms.^25^ If this is the case, then it may contribute to experience of fatigue. On the other hand, studies of induced visual stress have shown increased symptoms and an increased electromyography activity in the orbicularis oculi muscles.^28^ There are potentially both central and peripheral mechanisms contributing to the sense of fatigue after visually demanding tasks. The overall level of visual fatigue according to VAS score was lower at follow-up in our study, but the assessment session resulted in equal increase of visual fatigue at both baseline and follow-up. The visual assessment can be exhausting for patients. Some can get tired, suffer headaches and nausea, or even notice more visual symptoms afterward. A study using functional MRI showed that increased activation of visual processing increased self-reported perceived general fatigue in patients with MTBI.^29^ Self-reported perceived general fatigue may relate to visual processing and effort perception during the first months after MTBI.^8^,^29^ The feeling of reduced symptoms at the follow-up can eventually depend on the participants becoming used to and finding ways to cope with the disturbances. It might also be that the participants avoid doing visual things that make them uncomfortable.

The baseline evaluation showed that the participants commonly suffered from eye teaming defects (vergence) such as fusion at far and near, heterophoria, and vergence shift. Thiagarajan et al reported in a review article that MTBI patients suffer initially after the trauma from reading problems, vergence, version, and accommodation disturbances.^23^ In a recent systematic meta-analysis, Merezhinskaya and colleagues (2019) reported that CI increased almost in 40% in TBI.^30^ Pfeiffer and colleagues (2020) reported that CI correlated with high symptom burden in a recent MTBI diagnosis.^31^ General oculomotor dysfunction is common.^6^,^8^,^11^ A high prevalence of strabismus can be seen in the participants in both baseline and follow-up. MTBI can lead to worsening of preexisting heterophorias,^32^ and it might be that the hidden strabismus has not been detected or assessed before trauma even if existing. Many participants in this study suffered from eye movement dysfunctions at the initial stage, which also has been shown in the review by Thiagarajan and colleagues.^23^

The follow-up evaluation confirms, in line with previous studies, that the participants’ visual symptoms decreased for most participants. This is in line with previous studies.^7^–^9^,^23^-^25^,^30^ No spontaneous recovery was detected in vergence facility like fusion and accommodation functions.^18^ This also is in line with results from a recent study where about 40% of the participants with TBI still experienced persisting symptoms with accommodation and convergence 1 year after trauma.^33^

Almost all patients with DVA deficiency at baseline had a persistent deficiency at follow-up (7 out of 8 patients). Maintenance of the DVA requires an efficient orchestration of functions that control eye, head, and neck movements. Our findings show that persistent issues concerning eye movement control were common at follow-up. This study did not, however, account for cortical, cerebellar, cervical, or peripheral vestibular issues. Interventions targeting eye movement control and eye teaming may, however, be an important part of rehabilitation in the presence of persistent dynamic visual problems, postural imbalance, or ambulation problems.^34^,^35^

Clinical assessment of saccadic function in MTBI patients has previously not shown significant disturbances.^36^,^37^ This is equivalent with our findings using DEM ratio score. Ayton and colleagues found that DEM test performance does not correlate with saccadic eye movement skills or symptomatology but does relate to speed of visual processing and performance in reading.^38^ However, studies using eye tracking measures, which are more sensitive to changes in saccadic eye movement, have shown deviating results in patients with MTBI.^39^,^40^

Our study’s strengths are the well-defined study group and the use of comprehensive and standardized visual evaluations. The low sample size can be seen as a limitation in the study. The varying time span between baseline and follow-up can also be considered as a limitation. The patients’ wide age range, 24 to 66 years, might also be a limitation. Increasing age and female biological sex have been mentioned as risk factors for persistent disturbances.^24^ The group size in our study did not allow any analysis on these factors.

Previous research has shown that rehabilitation interventions aimed at improving visual function are linked to reduced symptoms,^41^,^42^ improved visual attention,^43^,^44^ and better performance in daily activities.^45^ A systematic review and meta-analysis study emphasizes that restitutive interventions may be beneficial for adults with oculomotor deficits after MTBI.^46^ Importance of evaluating both visual function and visual symptoms gives a broad understanding of visual difficulties associated to each other. Our results point out the importance of evaluation of visual disturbances after MTBI to provide visual therapy in case of persistent disturbances. For further studies, it would be an advantage to use eye tracking technology to find more sensitive markers to track visual deviations longitudinally.
